# Incorporating genetic and clinical data into the prediction of thromboembolism risk in patients with lymphoma

**DOI:** 10.1002/cam4.4280

**Published:** 2021-10-01

**Authors:** Mariana Bastos‐Oreiro, Javier Ortiz, Virginia Pradillo, Eduardo Salas, Carolina Marínez‐Laperche, Andrés Muñoz, Ismael Buño, José Luis Diéz‐Martin, Jose Manuel Soria, Cristina Pascual Izquierdo

**Affiliations:** ^1^ Hematology Department Hospital General Universitario Gregorio Marañon Madrid Spain; ^2^ Gregorio Marañón Health Research Institute Madrid Spain; ^3^ Genomics Unit, Hospital General Universitario. Gregorio Marañón Madrid Spain; ^4^ Cell Biology Department School of Medicine, Universidad Complutense de Madrid Madrid Spain; ^5^ Gendiag, S.L. Scientific Department Barcelona Spain; ^6^ Oncology Department Hospital General Universitario Gregorio Marañón Madrid Spain; ^7^ Hospital Universitario de la Santa Creu I Santa Pau Barcelona Spain

**Keywords:** genetic risk score, lymphoma complications, thromboembolism risk

## Abstract

**Background:**

The incorporation of genetic variables into risk scores for predicting venous thromboembolic events (VTE) could improve their capacity to identify those patients for whom thromboprophylaxis would be most beneficial. Proof‐of‐concept of this is provided by the TiC‐ONCO score for predicting the risk of VTE in patients with solid tumours. Our aim was to develop a similarly improved tool—the TiC‐LYMPHO score—for predicting VTE in patients with lymphoma.

**Methods:**

In a retrospective observational study of 208 patients with lymphoma, 31 (14.9%) were found to have experienced an episode of VTE either at the time of diagnosis or over the next 6 months. Clinical variables associated with VTE, determined via logistic regression analysis, plus the same genetic variables included in the TiC‐ONCO score, were used to build the TiC‐LYMPHO score algorithm. The sensitivity, specificity, predictive values and AUC of the TiC‐LYMPHO, the Khorana and ThroLy scores were compared in the same population.

**Results:**

The TiC‐LYMPHO score showed a significantly higher AUC, sensitivity and NPV (0.783, 95.35% and 97.98% respectively) than the other scores. The ThroLy score showed a significantly higher specificity (96.43% vs. 54.49%; *p* < 0.0001) and PPV (37.50% vs. 26.36%; *p* = 0.0147) than the TiC‐LYMPHO score, whereas its AUC, sensitivity and NPV were significantly lower (0.579, 19.35% and 86.48%, respectively).

**Conclusion:**

These results show that by incorporating genetic and clinical data into VTE risk assessment, the TiC‐LYMPHO score can categorize patients with lymphoma better in terms of their risk of VTE and allow individualized thromboprophylaxis to be prescribed.

## Graphical abstract

This is a single‐centre observational study that analyses the utility of combining genetic and clinical variables in a thromboembolism risk score in patients with lymphoma. The TiC‐LYMPHO score showed a significantly higher AUC, sensitivity and NPV (0.783%, 95.35% and 97.98%, respectively) than the other scores. These results show that by incorporating genetic and clinical data into VTE risk assessment, the TiC‐LYMPHO score can categorize patients with lymphoma better in terms of their risk of VTE and allow individualized thromboprophylaxis to be prescribed.

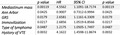

## 1 INTRODUCTION

Venous thromboembolic events (VTE) are a frequent complication in patients suffering from haematological malignancies, particularly lymphoma.^1^, ^2^ The incidence of such events is some 10%–15%, depending on whether all lymphomas are included or only those patients treated by chemotherapy.^1^, ^3^ In any event, they are a cause of increased morbidity and mortality^4^, ^5^ and thus represent an important economic cost to healthcare systems.^6^ They can be prevented by thromboprophylaxis, but this increases the risk of haemorrhage^10^, ^11^ and incurs additional treatment costs.^12^ It is therefore important to be able to identify those patients who can most benefit from such treatment.

Because the incidence of VTEs in cancer is high, it is important to evaluate the risk of their development at the time of diagnosis, and certainly before patients start chemotherapy.^6^ The risk of VTE associated with lymphoma has been assessed in multiple series of patients, focusing on clinical variables.^1^, ^2^, ^7^ In the most consolidated models, such as the Khorana score,^8^ lymphomas are considered to be a high risk factor for VTE. However, the usefulness of this score is limited; patients at high risk can still return an overall low‐middle score.^9^ Recently, a new and validated score—the ThroLy score—was proposed by Antic et al.^13^ for determining the risk of VTE in patients with lymphoma. However, this score too is based only on clinical and laboratory variables. It does not take into account any genetic factors. Our group also recently proposed a new score, the TiC‐ONCO score, for determining the risk of VTE in patients with solid tumours. This combines the clinical and genetic risk factors associated with VTE and was found to be significantly better than the Khorana score at identifying patients at high risk of VTE and who would therefore benefit from personalized thromboprophylaxis.^14^ The present work describes a new score—the TiC‐LYMPHO score, the algorithm of which was constructed taking into account the same kind of data as used in the TiC‐ONCO score—for determining the risk of VTE in patients with lymphoma.

## 2 PATIENTS AND METHODS

### 2.1 Study population

The study population of this observational, case–control study included all consecutive patients diagnosed with non‐Hodgkin (NHL) or Hodgkin lymphoma (HL) (based on the World Health Organization 2008 classifications^15^), presenting at the Gregorio Marañon University Hospital between January 2014 and December 2017. For the analysis, NHLs were grouped according to their clinical aggressiveness into indolent and aggressive. Aggressive lymphomas included diffuse large cell lymphoma, mantle lymphoma, and plasmablastic lymphoma and T lymphomas. Indolent lymphomas included follicular lymphoma, marginal lymphoma, lymphocytic lymphoma and lymphoplasmacytic lymphoma. Among the aggressive ones, a distinction was made between diffuse large cell lymphoma (DLBCL) due to its frequency. The same among the indolent, with follicular lymphoma (FL). Demographic, laboratory and clinical data were collected at diagnosis, and further clinical data and any VTEs recorded over the next 6 months.

### 2.2 Diagnosis of VTE

Deep vein thrombosis in the lower limbs was diagnosed by ultrasound or ascending venography. Pulmonary embolism was diagnosed by ventilation–perfusion lung scanning, pulmonary angiography or spiral computed tomography. Intracranial venous thrombosis was diagnosed by magnetic resonance imaging.

### 2.3 Thromboembolism risk variables

Data were collected on clinical and laboratory variables known to be associated with VTE risk.^16^, ^17^, ^18^, ^19^ These included lymphoma characteristics (histological type and subtype, stage, risk score, bulky disease, mediastinal localization and relapse/refractory disease), patient characteristics (age, sex, toxic habits, B symptoms, previous VTE, presence of a central venous catheter [port‐a‐cath in all cases in our series], family history of thrombosis, ECOG performance status, mobility and length of bed rest [days]) and laboratory results (blood cell counts, lactate dehydrogenase (LDH), fibrinogen value, activated partial thromboplastin time (APTT), International Normalized Ratio (INR) and C‐reactive protein). None of the patient was on prophylaxis therapy.

### 2.4 Sample genotyping

DNA was obtained from blood and bone marrow samples and genotyped in TaqMan assays using the EP1 Fluidigm platform (an efficient endpoint PCR system for high‐sample‐throughput SNP genotyping). Analyses were performed for F5 rs6025, F5 rs4524, F13 rs5985 and SERPINA10 rs2232698, i.e., the genetic variables included in the TiC‐ONCO score. Together these provide the genetic risk score (GRS) associated with VTE.^14^

### 2.5 Development of the risk model

We have counted with the previous experience in the generation of the clinical‐genetic VTE risk score, named TiC. TiC score for solid tumours included four genetic variants: rs6025, rs4524, 3 rs5985 and SERPINA10 rs2232698. In TiC, as clinical variables, it includes BMI > 25; family history of VTE and two variables linked to the tumour, the type of tumour and the TNM stage. For the generation of the score for patients suffering from lymphomas, we have used the same genetic variants. We have analysed whether BMI > 25, family history of VTE and some characteristics of the tumours could be useful to elaborate a VTE risk score in a similar way as in solid tumours. A risk model for VTE was constructed using the above genetic variables plus the clinical variables found to be significantly associated with the appearance of a VTE (*p* ≤ 0.25). All these variables were then subjected to multivariate logistic regression using an Akaike's Information Criterion based (AIC‐based) backward selection process, and those still associated with an increased risk of VTE were entered into the TiC‐LYMPHO score algorithm.

### 2.6 Comparison with other risk scores

The AUCs for the three scores were compared, along with the sensitivity, specificity and predictive values of each, taking into account their high risk cut‐offs, i.e., Khorana score ≥3,^6^ Thrombosis Lymphoma predictive score (ThroLy) >3^1^, ^3^ and that of the TiC‐LYMPHO as provided by the Youden J index.^20^

### 2.7 Statistical analysis

Continuous variables were recorded as means and categorical variables as proportions. Continuous variables were compared using the Student *t*‐test or the Mann–Whitney *U* test as required. Categorical variables were compared using the *χ* ^2^ or Fisher tests. The DeLong test was used to examine the differences between the AUCs. For cumulative incidence calculation, those patients who died, relapsed or developed a second neoplasm or develop a VTE were censored for. All calculations were performed using MedCalc Statistical Software v.18.11.3 (MedCalc Software bvba; https://www.medcalc.org; 2019).

## 3 RESULTS

**FIGURE 1:**
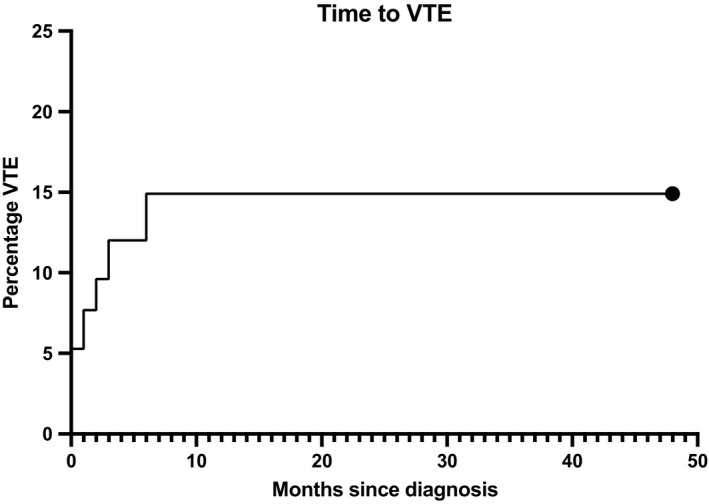
Cumulative incidence of VTE. At 6 months of follow‐up, the incidence of VTE was 14.9% (those patients who died, relapsed or developed a second neoplasm were removed from the analysis)

A total of 208 patients were diagnosed with lymphoma; over the study period, 31 (14.97%) of these experienced a VTE (Figure 1).

Aggressive lymphoma (AL) (diffuse large cell lymphoma, Burkkit's lymphoma, peripheral T lymphoma and mantle B cell lymphoma) was diagnosed in 125 patients (60%), Hodgkin lymphoma (HL) was diagnosed in 21 patients (10%) and indolent lymphoma (IL) (follicular B cell lymphoma and marginal B cell lymphoma) in 62 (30%). Sixty percent of the patients were in an advanced stage of disease (Ann Arbor III or IV), half of the patients had an extranodal localization (47%) and 50% received a central catheter (51%). The most common chemotherapy regimen used was R‐CHOP (Rituximab, cyclophosphamide, adramycin, vincristine and prednisone) or CHOP. CHOP was used in 119 patients (57%), mainly in those suffering from aggressive lymphomas (in 80% of such patients), followed by patients with indolent lymphoma (in 40% of these patients). Other treatments used for patients with AL were R‐EPOCH (Rituximab, Etoposide Phosphate, Prednisone, Vincristine Sulfate‐Oncovin‐, Cyclophosphamide and Doxorubicin Hydrochloride), CHOEP (Cyclosphosphamide, Doxorubicin Hydrochloride, Etoposide, Vincristine and Prednisone), Burkimab (Block A: Rituximab, Vincristine, Methotrewate, Iphosphamide, Dexamethason, Teniposide, Cytarabine; Block B, Rituximab, Vincristine, Methotrexate, Cyclophosphamide, Dexamethasone, Doxorubicin, Block C: Rituximab, Vindesine, Methotrexate, Dexamethasone, Etoposide and Cytarabine) and HyperCVAD (Course A: Cyclophosphamide, Vincristine, Doxorrubicin, Dexametasone, Cytarabine, Mesna, Methotrexate; Course B: Methotrexate, Leucovorin, Sodium bicarbonate and Cytarabine). Other regimen for IL was R‐Bendamustine. Ninety percent of patients with HL received ABVD (Adriamycin, Bleomycin, Vinblastine and Dacarbazine), and 10% received BEACOOP (Bleomycin, Etoposide, Doxorubicin hydrochloride, Cyclophosphamide, Vincristine, Procarbazine and Prednisone). All patients with AL developed neutropenia during treatment.

Table 1 shows the clinical characteristics of the patients who experienced or did not experience a VTE. Univariate regression showed bed rest for >3 days, and having a central venous catheter, a ThroLy score of ≥2, a family or personal history of VTE, an advanced Ann Arbor stage (IV), a cancer histological type of aggressive DLBCL, non‐DLBCL or PCNSL and the presence of a mediastinal mass to be more common in patients who experienced a VTE. These patients also showed a trend towards having B symptoms. Patients who did not experience a VTE more commonly had an FL or an indolent LNH histological type.

**TABLE 1:** Clinical characteristics for lymphoma patients with or without VTE, expressed as *N* (%)

	VTE	No VTE	*p* value
	31 (19.9)	177 (85.10)	
Age, years±SD	68.00 ± 16.64	60.24 ± 18.31	0.0173
Male *N* (%)	20 (64.52)	91 (51.41)	0.1779
BMI			
<1 8.5	2 (6.45)	4 (2.26)	0.2
18.5–24.99	11 (35.48)	54 (30.50)	0.6
25–29.99	11 (35.48)	58 (32.77)	0.8
>30	7 (22.58)	20 (11.30)	0.08
Unknown	0 (0)	41 (23.16)	0.0029
Ann Arbor			
I	3 (9.68)	9 (5.08)	0.4
II	5 (16.13)	20 (11.3)	0.4
III	6 (19.35)	15 (8.47)	0.06
IV	14 (45.16)	27 (15.25)	0.0001
Bed rest			
Yes	16 (51.61)	39 (22.03)	0.0006
No	15 (48.39)	138 (77.97)	
Central venous catheter			
Yes	22 (70.97)	87 (49.15)	0.02
No	9 (29.03)	90 (50.85)	
History of VTE			
Yes	9 (29.03)	16 (9.04)	0.002
No	22 (70.97)	161 (90.96)	
Histological type			
HL	5 (16.13)	17 (9.60)	0.3
FL	3 (9.68)	49 (27.68)	0.03
Indolent NHL	1 (3.23)	38 (21.47)	0.02
DLBCL	13 (41.94)	50 (28.25)	0.13
Aggressive, non DLBCL	7 (22.58)	22 (12.43)	0.13
Aggressive non DLBCL + non DLBCL (total)	20 (63.52)	72 (40.68)	0.04
PCNSL	2 (6.45)	1 (0.56)	0.01
B Symptoms			
Yes	17 (54.84)	64 (36.16)	0.05
No	14 (45.16)	113 (63.84)	
Bulky disease			
Yes	10 (32.26)	37 (20.90)	0.2
No	21 (67.74)	140 (79.10)	
Extranodal localization			
Yes	14 (45.16)	84 (47.46)	0.8
No	17 (54.84)	93 (52.54)	
Mediastinum mass			
Yes	6 (19.35)	12 (6.78)	0.02
No	25 (80.65)	165 (93.22)	
Khorana score ≥3			
Yes	2 (6.45)	10 (5.65)	0.9
No	29 (93.55)	167 (94.35)	
ThroLy score ≥2			
Yes	21 (67.44)	54 (30.51)	0.0001
No	10 (32.26)	123 (69.49)	

Non‐Hodgkin lymphoma (NHLs) were grouped according to their clinical aggressiveness into indolent and aggressive. Aggressive lymphomas included diffuse large cell lymphoma (DLBCL), mantle lymphoma, and plasmablastic lymphoma and T lymphomas. Indolent lymphomas included follicular lymphoma, marginal lymphoma, lymphocytic lymphoma and lymphoplasmacytic lymphoma. Among the aggressive ones, a distinction was made between diffuse large cell lymphoma (DLBCL) due to its frequency. The same among the indolent with follicular lymphoma (FL).

### 3.1 Variables included in the TiC‐LYMPHO algorithm

After multivariate regression, the genetic variables included in the TiC‐ONCO score,^14^ plus the following clinical variables, were included in the TiC‐LYMPHO algorithm: the type of lymphoma according to the WHO classification,^21^ mediastinal involvement, Ann Arbor stage, bed rest for >3 days, and a family or personal history of VTE. Table 2 shows the variables independently associated with the risk of VTE in the multivariate analysis. TiC‐LYMPHO algorithm can be used at the time of diagnosis of the lymphoma. The use of this score considering the Jouden index J as a cut‐off will identify if any particular subject suffering from lymphoma is at high risk or at a low risk of developing a VTE in the 6 months after the diagnosis.

**TABLE 2:** Multivariable analysis

	*p* value	HR	95% CI	*p* value
Mediastinum mass	0.00119	4.5562	1.1091–18.7174	0.00119
Ann Arbor	0.0425	0.9007	0.7312–0.9994	0.0425
GRS	0.0179	2.6581	1.1161–6.3309	0.0179
Immovilization	0.0217	2.6856	1.0519–6.8566	0.0217
Type of lymphoma	0.0487	1.2175	1.0505–1.7497	0.0487
History of VTE	0.0032	4.1622	1.4598–11.8674	0.0032

Variables independently associated with VTE risk in lymphoma patients at diagnosis.

### 3.2 Comparison of the different scores

Table 3 shows the discriminative and predictive capacity of the different scores. The TiC‐LYMPHO score showed a better AUC than both the ThroLy and Khorana scores (0.783 vs. 0.579 and 0.502, respectively; *p* < 0.0001 for both). The TiC‐LYMPHO score also showed higher sensitivity (93.55% vs. 19.35% and 6.45% for the ThroLy and Khorana scores, respectively; *p* < 0.0001), the best negative likelihood ratio (LHR‐) (0.12 vs. 0.47 and 0.86.1, respectively; *p* < 0.0001) and the best negative predictive value (NPV) (97.98% vs. 86.48%, and 84.41%, respectively; *p* < 0.0001). The positive predictive value (PPV) shown by the TiC‐LYMPHO score was significantly better than that of the Khorana score (26.36% vs. 16.67%; *p* = 0.0161), but lower than that of the ThroLy score (26.36% vs. 37.50%; *p* < 0.0001). Similarly, the LHR + shown by the TiC‐LYMPHO score was significantly better than that shown by Khorana score (2.06 vs. 1.39; *p* < 0.0001), but lower than that shown by the ThroLy score (2.06 vs. 3.29; *p* < 0.0001). Finally, the specificity of the TiC‐LYMPHO score was significantly lower than that of both the ThroLy and Khorana scores (54.49% vs. 96.43% and 94.01%; *p* < 0.0001).

**TABLE 3:** Sensitivity, specificity, positive predictive value (PPV), negative predictive value (NPP), area under the curve (AUC) and confidence interval 95% (95%CI)

	Sensitivity	Specificity	PPV	NPV	AUC	95% CI	*p* value
TiC‐LYMPHO	93.55	54.49	26.36	97.94	0.78	0.7231–0.837	0.0001
Khorana	6.45	94.01	16.60	84.41	0.503	0.431–0.574	0.902
ThroLy	19.35	96.43	50	86.63	0.57	0.50–0.648	0.0319

Treatment with semuloparin^22^ of all those patients with a TiC‐LYMPHO score indicating them to be at high risk (63.64%) would have prevented 58% of the VTEs recorded. The treatment of all the patients with a Khorana score indicating the same would have prevented 6.45%, and the treatment of all those with a positive ThroLy score would have prevented 9.68%.

## 4 DISCUSSION

The present results show the TiC‐LYMPHO score to be better than the Khorana and ThroLy scores at identifying patients with lymphoma who are at high risk of VTE. The incidence of VTE in patients with solid malignancies and blood cancers is high.^23^ In patients with lymphoma, it is no less important to determine—at the time of diagnosis—the risk of experiencing a VTE and only then can adequate preventive steps be taken.^4^, ^24^ When the risk of VTE is not known, the use of prophylaxis in patients with lymphoma is often restricted due to the fear of haemorrhage or of thrombopenia during chemotherapy. This, however, may be overcautious since thrombopenia at diagnosis is uncommon, even in aggressive lymphomas,^25^, ^26^ and chemotherapy does not often cause thrombopenia.^27^, ^28^ In the last decade, multiple randomized clinical trials, including in patients with lymphoma,^29^ have shown the efficacy and safety of oral and parenteral thromboprhophylaxis^30^ in the ambulatory setting.

The Khorana score^8^ is certainly one of the most commonly used risk scores for predicting VTE in patients with cancer. However, it is of limited use in lymphoma,^31^ probably because it understands it as a single entity, with no distinction among different histologies, tumour burdens or locations.^31^ The ThroLy score is a new score for assessing VTE risk in patients with lymphoma, which classifies them as being at low, intermediate and high risks.^13^ The variables included in this score are previous venous and/or arterial events, mediastinal involvement of lymphoma, BMI >30, reduced mobility, extranodal disease, neutropenia and a haemoglobin concentration of <100 g/L. However, it does not take into account any genetic variable. This model shows a PPV of 65.2% for identifying patients at high risk for VTE (4/7 points or more). Although this has been validated, it is important to note that there are also studies that show its accuracy to be limited in patients with lymphoma.^18^, ^32^ Indeed, the present results highlight the inaccuracy of both the Khorana and ThroLy scores. For example, the Khorana score showed a non‐significant AUC (*p* = 0.503) and very poor sensitivity (6.45%), and for a value of ≥4 the ThroLy score showed a low AUC (albeit statistically significant at *p* = 0.032) and identified just 6 of the 31 patients who experienced a VTE. It also had a sensitivity of just 19.35%. Based on these results, the ThroLy score would not have predicted 80% of the VTEs recorded. Moreover, of the 12 patients with a ThroLy score of ≥4, only six experienced a VTE (PPV 50%). However, the test did identify 162 of 168 with no VTE (specificity 98.43%). Similarly, of the 187 patients with a ThroLy score of ≤1, just 25 went on to experience a VTE (NPV 86%). The main objective of the application of these scores should be to reduce the VTE, and it is clear that this is not adequately achieved with the scores available so far. This is probably the reason why these scores have not been incorporated into management guidelines for lymphoma patients.

The proposed TiC‐LYMPHO score identified 29 of the 31 patients who experienced a VTE (sensitivity 93.55%). Had this result been used to guide the prescription of prophylactic treatment, only 6.4% of the patients who had needed prophylaxis would not have received it. A high‐risk TiC‐LYMPHO score identified 97 of the 178 patients who did not experience a VTE (PPV 54.49%); thus, part of the present population would have been over‐treated. However, almost all patients at risk can be given prophylaxis safely as long as they are adequately monitored. Considering the comorbidities that a VTE generates in this type of patients, requiring admission, anticoagulation at full doses and taking into account a difficult management since many of these patients frequently suffer thrombopenia associated with chemotherapy, we understand that the benefit of identifying the vast majority of patients at risk is greater than the harm of overtreating with low doses of heparin. Despite the extended idea that the administration of profilactic doses of anticoagulants is dangenous for patients with cancer, especially with haematological cancer, published reviews teach us the contrary. Herishau Y et al. published a review^33^ showing that even during the period of severe thrombocytopenia induced by intensive chemotherapy in pateints with haematological malignances, the administration of reduced doses of enoxaparin exerted protective capacity with no major bleeding effects.

From a clinical point of view, the present results show the TiC‐LYMPHO score to be more useful than the other two scores for guiding the prescription of thromboprophylaxis. The treatment of all the patients with a TiC‐LYMPHO score indicating high risk would have prevented 58% of all the VTEs recorded. The treatment of all the patients with a Khorana score indicative of high risk would only have prevented 6.45%, and the same for the ThroLy score where it is only 9.68%.

In summary, the predictive power of the TiC‐LYMPHO score was found to be significantly greater than that of the Khorana and ThroLy scores. This superiority is demonstrated by a better AUC, better likelihood ratios, a higher PPV and NPV and, importantly, a much higher sensitivity (93%).

## 5 CONCLUSIONS

The incorporation of genetic and clinical variables associated with thrombosis into the TiC‐LYMPHO score allows the risk of VTE in patients with lymphoma to be much better identified, offering the opportunity to prescribe individualized thromboprophylaxis. The present results also contribute to the validation of the TiC‐ONCO score.

## CONFLICT OF INTEREST

Authors declare no conflict of interest.
